# Early Implementation of a Patient-Centered Medical Home in Singapore: A Qualitative Study Using Theory on Diffusion of Innovations

**DOI:** 10.3390/ijerph182111160

**Published:** 2021-10-24

**Authors:** Zoe Zon Be Lim, Mumtaz Mohamed Kadir, Mimaika Luluina Ginting, Hubertus Johannes Maria Vrijhoef, Joanne Yoong, Chek Hooi Wong

**Affiliations:** 1 Geriatric Education and Research Institute, Singapore 768024, Singapore; mohamedkadir.mumtaz@geri.com.sg (M.M.K.); ginting.mimaika.luluina@geri.com.sg (M.L.G.); jyoong@usc.edu (J.Y.); wongchekhooi@tsaofoundation.org (C.H.W.); 2 Panaxea, 1098 XH Amsterdam, The Netherlands; Bert.Vrijhoef@panaxea.eu; 3 Maastricht University Medical Center, 6229 HX Maastricht, The Netherlands; 4 Center for Economic and Social Research, University of Southern Carolina, Los Angeles, CA 90089, USA; 5 Research for Impact, Singapore 159964, Singapore; 6 Tsao Foundation, Singapore 168730, Singapore; 7 Health Services & Systems Research, Duke-NUS, Singapore 169857, Singapore

**Keywords:** Patient-Centered Medical Home, primary care, innovation, implementation, complex needs, adoption, assimilation

## Abstract

Patient-Centered Medical Home (PCMH) has been found to improve care for complex needs patients in some countries but has not yet been widely adopted in Singapore. This study explored the ground-up implementation of a PCMH in Singapore by describing change strategies and unpacking initial experience and perception. In-depth interviews were conducted for twenty-two key informants from three groups: the implementers, their implementation partners, and other providers. “Diffusion of innovations” emerged as an overarching theory to contextualize PCMH in its early implementation. Three core “innovations” differentiated the PCMH from usual primary care: (i) team-based and integrated care; (ii) empanelment; and (iii) shared care with other general practitioners. Change strategies employed to implement these innovations included repurposing pre-existing resources, building a partnership to create supporting infrastructure and pathways in the delivery system, and doing targeted outreach to introduce the PCMH. Initial experience and perception were characterized by processes to “adopt” and “assimilate” the innovations, which were identified as challenging due to less predictable, self-organizing behaviors by multiple players. To work with the inherent complexity and novelty of the innovations, time, leadership, standardized methods, direct communication, and awareness-building efforts are needed. This study was retrospectively registered (Protocol ID: NCT04594967).

## 1. Introduction

A robust primary care (PC) system is the foundation to a high-functioning health system and healthy populations [1,2]. With the aging of the population, there are increasing pressures for PC to manage older, chronically ill patients with complex needs such as multimorbidity, geriatric syndromes, co-existing cognitive impairments, mental illness, or psychosocial vulnerabilities [3,4,5]. Complex chronic patients require the provision of care that is person-centered, comprehensive, coordinated, and longitudinal. These requirements are often unmet by PC practices that lack adequate training, multidisciplinary care teams, appropriate practice infrastructure, and payment systems [5,6,7]. Without good-quality PC, complex chronic patients experience worse health outcomes and consume higher proportions of healthcare services and costs [8,9]. Hence, PC reforms are imperative to address the service gaps for complex chronic patients [10,11]. However, it is well known that PC reforms are time-consuming, costly, and unpredictable in their outcomes. Without appropriate change strategies, the possibility of failure, burnout, and financial losses are real for PC providers participating in these reforms [12,13].

New PC models have been trialed and one model gaining popularity in recent years is the Patient-Centered Medical Home (PCMH). PCMH is a PC redesign whereby patient care is delivered through a centralized setting to enable “care integration, family and patient partnership and engagement, and operationalization of the PC core attributes of personal, first-contact access, comprehensive, and coordinated care” [14,15]. Typically, practice transformation to a PCMH model involves the addition or modification of existing infrastructure and/or workflow (e.g., care managers, clinical information system). Furthermore, some PCMHs also integrate with the mental health or social services in the delivery systems they reside [16]. Practice transformation is context-dependent and there is no one-size-fits-all model for PCMHs [15,17]. Nonetheless, all PCMHs share the core feature of a team-based structure (with two or more clinicians working together), and other features that operationalize the PCMH core principles of (i) enhanced access (e.g., after-hours care); (ii) coordinated care (e.g., good care plan transitions); (iii) comprehensiveness (e.g., integrated psychosocial care services); (iv) patient-centeredness (e.g., active engagement of patients and family members); and (v) quality and safety (e.g., use of clinical information technologies) [18,19]. PCMHs have demonstrated significant improvements in episodes of depression, health-related quality of life, self-management outcomes, and biomedical markers such as blood pressure and glycated hemoglobin [20]. They have also improved patient experience, mitigated disparities, reduced utilization of acute care and overall healthcare expenditure [21]. PCMH has been recommended as a model for PC reforms and systematically disseminated in the US and Australia [22,23]. However, there is a paucity of evidence for implementing PCMHs in other countries.

This qualitative study explored the early implementation of a new PCMH care model in Singapore. It was part of a larger research project that aimed to evaluate the impacts of the PCMH intervention on patient experience, quality of life, patient activation, healthcare utilization, and costs. Findings on the patient-reported outcomes have been published elsewhere [24]. This study adds to the larger research by providing an understanding of the implementation, through three research questions:

1. What were the change strategies employed for implementing a new PCMH care model in Singapore?
2. How were the PCMH and its change strategies experienced and perceived?
3. What were the lessons learned?

**Figure 1:**
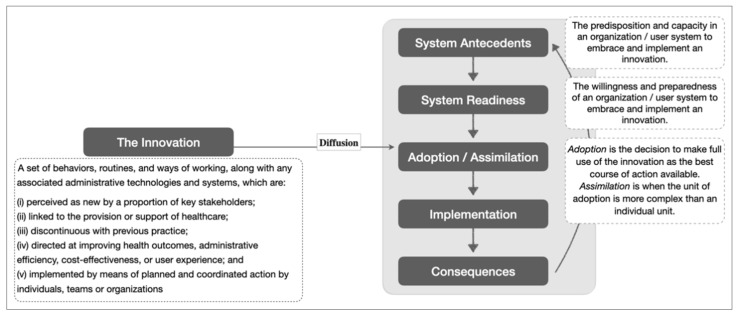
Simplified theoretical framework on the diffusion of innovation with definitions for its key constructs. Adapted from [25].

An exploratory approach was employed due to the novelty of this care model to the Singapore health system. This led to the use of a theoretical framework on the diffusion of innovations developed by Greenhalgh and colleagues to contextualize implementation experience. The theory explains different components and their interactions that influence the diffusion of new ideas, products, services, etc. [25]. For the purpose of this study, we focused on certain components of the theoretical framework, namely “system antecedents”, “system readiness” and “adoption/assimilation” (see Figure 1).

## 2. Primary Care Reform and Early Adoption of Patient-Centered Medical Home in Singapore

In Singapore, PC reforms have been underway in response to rapid population aging. By 2030, one in four Singaporeans will be aged 65 and above [26]. In a population study conducted in 2016–2017, nearly 40% of Singaporeans aged 60 and above had three or more chronic illnesses [27]. The burden of increasing chronic illness demands that chronic care be delivered in the community, in a manner that is comprehensive, continuous, person-centered, and integrated or coordinated with other service providers [28,29].

At present, Singapore’s health system is divided into three integrated clusters known as Regional Health Systems (RHSs), each comprising a network of public-sector healthcare providers [30]. The usual PC in Singapore is provided by either public polyclinics or private practices in the community (Box 1). PC providers are usually led by physicians, with very few practicing multidisciplinary team-based care. Patients may utilize more than one PC provider of their choice. Chronically ill patients with complex needs are usually referred to specialist outpatient clinics (SOCs) in acute hospitals [31,32]. If referrals are made by polyclinics to SOCs in public hospitals, patients are eligible for RHS-based subsidies. Therefore, both polyclinics and public hospitals often have high patient volumes. Shared care between PC providers and SOCs is an emerging trend but still uncommon. Meanwhile, shared care across individual PC providers is highly limited, to the best of our knowledge [33].

In more recent years, PC reforms have been initiated to improve readiness to deliver comprehensive, team-based care in the community. There has been an increasing trend in the development of partnerships among various providers to test out region-specific innovations for potential national-level dissemination [34]. Examples of PC innovations implemented at the national level include the redesign of private practices into team-based Family Medicine Clinics led by family physicians, the creation of Community Health Centers to provide support services to private general practitioners (GPs), the introduction of team-based care in the polyclinics, development of shared services for GPs through the Primary Care Network, and the integration and co-location of health and social services for older adults in one-stop centers called Geriatric Service Hubs [33,35].

Into this landscape, the PCMH model was introduced by a private GP group, two acute hospitals, and a non-profit family foundation [36,37]. However, relatively little has been published about these pioneering PCMH initiatives. One study evaluated patient experience [38], while another study examined implementation challenges, which included high setting-up costs, unfamiliarity with new care models, and restrictions in utilizing RHS-based subsidies [13].

### About ComSA-Patient-Centered Medical Home

Launched in November 2016, ComSA-PCMH is a new PC model in Singapore co-developed by the central-region RHS and Tsao Foundation (TF). The foundation is experienced in providing primary medical and psycho-social care for community-dwelling older adults. ComSA-PCMH is part of “ComSA” (Community for Successful Ageing), which is a community-wide aging-in-place initiative in Whampoa where there is a relatively high proportion of older persons (18% aged 60 or above out of a population of 30,500, 2015 figure) [39]. In a 2014 community needs survey of 1,325 older Whampoa residents aged 60 or above, participants reported risk of cognitive impairment (10%), social isolation (53%), and inappropriate help-seeking behaviors (25.6%) [40]. Box 1 summarizes the context-specific acronyms and terminology used in this study.

ComSA-PCMH was established to serve a target population of Whampoa residents aged 40 or above identified to have complex medical and psychosocial needs. Such targeted service is called “empanelment”, which is defined as “a continuous, iterative set of processes that identifies and assigns populations to facilities, care teams, or providers who have a responsibility to know their assigned population and to proactively deliver coordinated primary health care towards achieving universal health coverage” [41]. It has two distinct but integrated care components, i.e., a PC clinic and home-based care management (CM) service. The PC clinic is led by family physicians trained in geriatric medicine and care coordination. Physicians are supported by registered nurses and a clinic executive (information and referral). As patients have complex needs, the duration of the consultation is typically longer. First visits can last up to sixty minutes to allow sufficient time for doctor-patient communication, comprehensive medical and psychosocial assessments, and health education. Patients with higher psycho-socio-behavioral needs are referred to an integrated CM service, which is home-based. Care managers assess patients’ home environment and their broader support system. They also address financial and socio-behavioral issues, coordinate care with relevant social service providers, and ensure social support is sustainable before they exit. The CM service is led by social workers and nurse care managers, with the support of assistant care managers and a program coordinator.

Box 1 **Terminology Used in This Study.**

CM (care management): is a home-based service offered to patients with complex psychosocial needs. Integrated with a PC clinic, they formed ComSA-PCMH. ComSA (Community for Successful Aging): is a community-wide initiative in the Whampoa precinct, which provides an integrated system of programs and services with the aim of promoting health and wellbeing over the life course, and enabling aging in place [42]. ComSA-BPS-RS (ComSA BioPsychoSocial Risk Screener): is a risk screening tool purpose-built for ComSA and locally validated in 1107 community-dwelling older adults aged 60 or above in Whampoa. It contains 37 questions from biological, psychological, and social dimensions, with each dimension having equal weightage to a final score that stratifies older adults into four categories: i.e., “managing well” (Category 1); having “some problems” (Category 2); “many problems” (Category 3); “overwhelming problems” (Category 4) in managing their health [43]. ComSA-PCMH (ComSA-Patient-Centered Medical Home): is the integrated service of two key services (i.e., PC clinic and home-based CM service). It is the PC innovation under investigation by this study. Polyclinics: are the public-sector PC providers in Singapore. They provide medical treatment, preventive healthcare, and health education at a subsidized cost. There are twenty polyclinics that cater to 20% (2014 figure) of PC clinic attendances in the country [35,44]. Private GPs (General Practitioners): are the private-sector PC providers in Singapore, who work in either solo or group practices. Services offered to vary according to the strengths of GPs and local needs. There are about 1700 Private GPs that cater to 80% (2014 figure) of PC clinic attendances in the country [35,44]. RHS (Regional Health System): is an integrated cluster of public healthcare providers including acute hospitals, polyclinics, and community hospitals. There are currently three RHSs (Central, Eastern, and Western regions) in Singapore. The central-region RHS was the implementation partner of ComSA-PCMH. TF (Tsao Foundation): is the non-profit family foundation that initiated and implemented ComSA-PCMH, in partnership with the central-region RHS. The foundation has more than 25 years of experience serving community-dwelling older persons in Singapore.

## 3. Methods

### 3.1. Study Design and Sampling

The study employed a predominantly grounded theory approach, which is a methodology that constructs theory based on emerging patterns from empirical data [45], to explain patterns of behavior observed in the early implementation of a new care model. Semi-structured, in-depth interviews (IDIs), which allow exploration of an individual’s unique perspective, were employed to collect empirical data.

Study participants consisted of three groups: (i) “implementers” from TF; (ii) “implementation partners” from the RHS; and iii) other PC providers in Whampoa, predominantly the private GPs. Within groups (i) and (ii), participants were purposely sampled from both managerial vs. non-managerial, as well as clinical (e.g., doctors, nurses, social workers) vs. non-clinical (e.g., clinic executive) positions, to give a diverse range of organizational, professional, and individual perspectives. These participants had at least three months of experience interacting with ComSA-PCMH. Group (iii) did not necessarily need to have interacted with ComSA-PCMH to be recruited into the study.

This study was retrospectively registered with ClinicalTrials.gov (Protocol ID: NCT04594967).

### 3.2. Data Collection

Thirteen implementers and seven implementation partners were interviewed between November 2017 and June 2018. Two private GPs were interviewed about a year later (June–October 2019), as more time was needed for GPs to learn about ComSA-PCMH. Lastly, two additional implementers were interviewed in July 2020 for theoretical saturation (see Section 3.3). Interview topic guides were developed using PCMH principles defined by the Agency for Healthcare Research and Quality [19], with additional topics on practice transformation and perceived outcomes (Supplementary File S1). A key question asked was “How would you describe your experience in implementing (a certain component of) ComSA-PCMH?”. The objective of the IDIs was to allow participants to freely express their interpretations about the challenges and facilitators experienced during early implementation. IDIs were between 45 to 90 min, audio recorded, and transcribed verbatim. Participants were interviewed only once within the initial 3.5 years of the innovation. A small token of appreciation was given at the end of the IDIs.

### 3.3. Data Analysis

IDI transcripts were initially coded based on the PCMH-related topics in the interview guide. However, this approach was found to be inadequate to explain some emerging patterns in the codes. The analysis then shifted to a grounded theory approach, which involved three iterative steps: inductively detecting new patterns from the data, finding suitable theoretical frameworks in the literature, and in-depth discussion between two analysts who are experienced in qualitative analysis (ZZBL and MMK). After multiple rounds of data-framework-discussion process, analysis arrived at the theoretical framework on the diffusion of innovations by Greenhalgh and colleagues [25]. Constructs in the framework (Figure 1) were used to re-organize codes, explain the relationship between categories (i.e., clusters of related codes), and provide insight into the emerging themes [46,47]. Figure 3 is an example showing the relationship between codes, categories, and themes, with supporting theory. To ensure theoretical saturation, all transcripts were re-coded based on this new theoretical framework and two additional interviews were conducted. About a third of the transcripts were double coded to ensure intercoder reliability. Both analysts were also in a unique position of having worked in TF prior to data analysis and were exposed to the organizational culture and the day-to-day implementation of ComSA-PCMH. This additional ethnography-like experience offered a depth of tacit knowledge, which was used for elucidating the nuances in the data. Potential implicit biases were uncovered through reflexivity and mutually challenging each other’s views [48,49]. Using Nvivo (v.12) (QSR International, Doncaster, Australia) and the comment function in Microsoft Word, an audit trail was kept for codes, categories, emerging themes, and discussions made to resolve differences in interpretations. Findings were also presented to other members of the study team, advisors, implementers, and implementation partners for the enhancement of interpretations. The Standards for Reporting Qualitative Research (SRQR) was used to guide the reporting of findings.

## 4. Results

**Figure 2:**
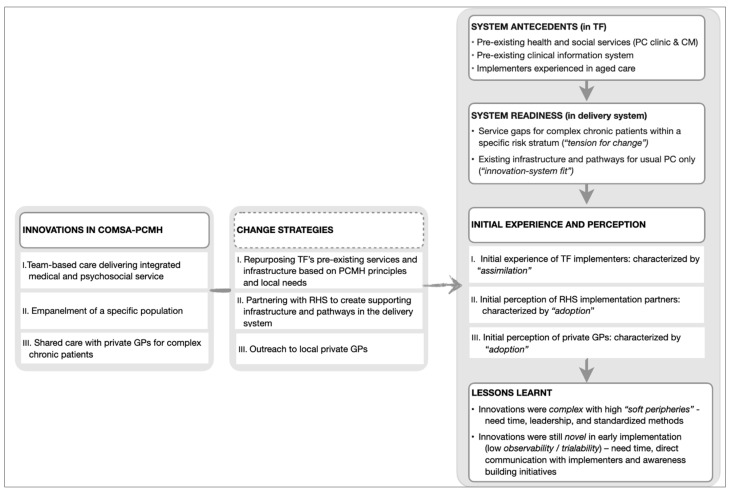
Key themes for Innovations, Change Strategies, Initial Experience and Perception, and Lessons Learnt. TF: Tsao Foundation; RHS: Regional Health System; GPs: General Practitioners; PC: Primary Care; CM: Care Management.

Diffusion of innovations—which is a theory explaining the process experienced by individuals when adopting new ideas, products, services, etc.—emerged as the overarching theory to contextualize ComSA-PCMH’s early implementation. Results are presented in three parts, following three core “innovations” which emerged from the data, namely: (i) team-based and integrated care; (ii) empanelment; and (iii) shared care with other GPs. These “innovations” were identified from codes that matched the five defining characteristics of innovation in health service organizations: i.e., related to healthcare services or support, differentiated from usual practice, perceived as new by key stakeholders, aim to improve certain outcomes related to patient care, and led by strategized implementation (Figure 1). Each of the core innovations was identified alongside its respective change strategy and participants’ initial experience and perception when encountering the innovation. “Adoption” and “assimilation” emerged as the key themes for explaining the initial experience and perception of the innovations. Figure 2 summarizes these findings and lessons learned from the early implementation of ComSA-PCMH.

### 4.1. Innovation I. Team-Based Care Delivering Integrated Medical and Psychosocial Services

The first innovation differentiating ComSA-PCMH from the usual PC was team-based care that aimed to deliver integrated medical and psychosocial services (vs. medical care only).

#### 4.1.1. Change Strategy: Repurposing TF’s Pre-Existing Services and Infrastructure Based on PCMH Principles and Community Needs

TF had pre-existing services which cared for older patients with complex chronic needs, hence ComSA-PCMH was not entirely new to the implementers (Quote 1, see Table 1). Two services (i.e., a PC clinic and a home-based CM service) were repurposed to become one integrated team. Strategies supporting the integration between these services included: (i) co-location of PC clinic and CM workspace; (ii) redesign of roles and workflow towards team-based care; and (iii) communication to facilitate team building or team-based decision-making (e.g., interdisciplinary meetings, team huddles) (Quote 2). Services were also redesigned to suit community needs. For example, more clinical time was spent on managing family dynamics as most older persons in Whampoa were living with families. This differed from pre-existing services which addressed the needs of another community with more older persons living alone (Quote 3). In addition, clinical information systems were enhanced to remove the need for double entries of health records and billing information, facilitate team-based communication and patient tracking. Implementers were upskilled in the provision of aged care, care coordination, patient-centered and team-based care, through on-the-job training by experienced peer implementers and sharing from external experts.

#### 4.1.2. Initial Experience of TF Implementers: Characterized by “Assimilation”

**Figure 3:**
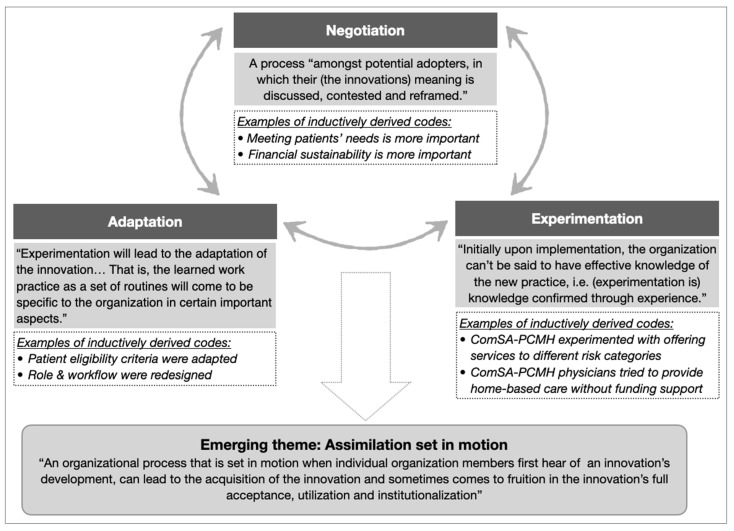
The theme of assimilation emerged from the cyclical relationships between three categories (negotiation, experimentation, and adaptation). Categories were developed from clusters of related codes (examples in italics). Supporting theory (in shaded boxes, adapted from [25,50]) was used to identify categories and emerging themes.

TF implementers experienced their new or redesigned roles and workflow in a complex, non-linear, and iterative manner, best described as a cyclical process of “experimentation”, “negotiation”, and “adaptation”. This process pointed to an emerging theme of “assimilation” (Figure 3), which characterized the complexities experienced by implementers when carrying out the change strategies to collectively adopt the innovation.

Example 1 of assimilation: Defining “complex needs”

- Context: The ComSA BioPsychoSocial Risk Screener (ComSA-BPS-RS) was developed to provide operational definitions for complex needs (Box 1). Patient eligibility was determined based on ComSA-BPS-RS categories.
- Negotiation: Implementers had to negotiate about different views on patient eligibility criteria. Initially, only patients in the two highest-risk categories of ComSA-BPS-RS (i.e., having “many problems” or “overwhelming problems” in managing their health risk) were eligible for receiving care from ComSA-PCMH, because ComSA-PCMH was perceived to be a resource-intensive model best reserved for patients with the highest needs (Quote 4). Over time, some implementers advocated for patients in the next category (i.e., having “some problems”) to also receive care to prevent their deterioration to the highest risk category.
- Experimentation: Some patients from the “some problems” category started to receive care from ComSA-PCMH.
- Adaptations: Eligibility criteria were revised to include patients in the “some problems” category, in alignment with the philosophy of providing care in a proactive manner (Quote 5).

Example 2 of assimilation: Introducing home medical service for homebound patients

- Context: There was a lack of home medical service to meet the demand of a rising number of homebound patients in Whampoa. However, funding for ComSA-PCMH did not account for this service.
- Negotiation: There were two emergent perspectives that had to be negotiated. The first perspective was that ComSA-PCMH (as a patient-centered care model) should prioritize meeting all patients’ needs, while the other perspective was that it would be financially unsustainable for physicians to provide home-based care (Quote 6).
- Experimentation: ComSA-PCMH physicians conducted home visits to provide home-based care in the absence of supporting funding (Quote 7).
- Adaptations: A new home medical program was introduced by TF, and workflow in ComSA-PCMH was redesigned to coordinate care with this new program (Quote 8).

**Figure 4:**
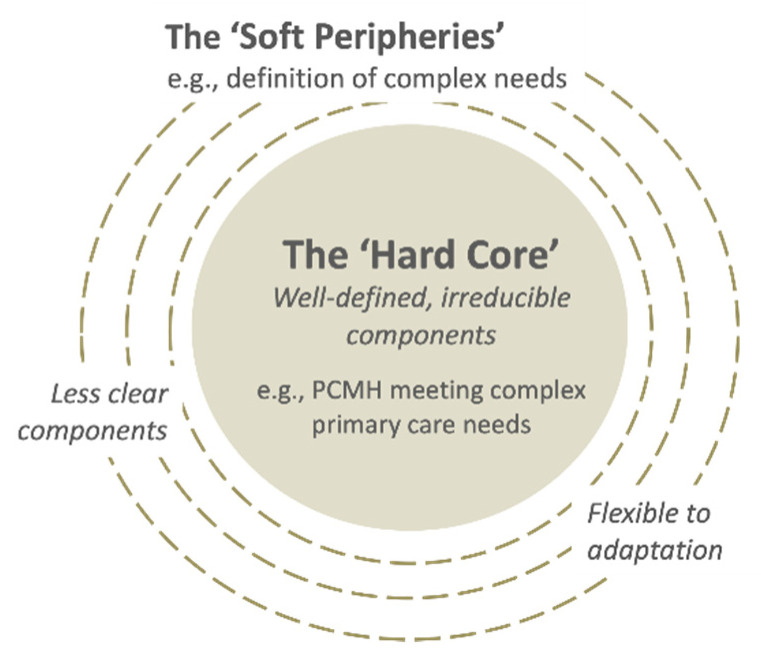
The “hard core” and the “soft peripheries” in a complex innovation. Adapted from [25].

Assimilation was perceived as challenging because of: (i) implementers’ multiple perspectives (Quote 9); and (ii) high number of “soft peripheries”, or the less clear and adaptable components of the innovation (Figure 4) (Quote 10), as shown in the two examples above. Assimilation was also hindered in cases of perceived lack of leadership in decision-making (Quote 11) and slow progress in transforming the clinical information systems (Quote 12). On the other hand, implementers’ capacity for assimilation was facilitated by their intrinsic motivation to contribute to aged care (Quote 13), relevant competencies and/or training in patient-centered and integrated care (Quote 14), willingness to experiment, and observability of the impacts from their invested efforts (Quote 15).

### 4.2. Innovation II. Empanelment of a Specific Population

The second innovation was empanelment, which allowed ComSA-PCMH to proactively deliver integrated PC services to its target population, i.e., those (i) aged 40 or above; (ii) living in Whampoa precinct; and (iii) residing in the risk stratum which requires a higher level of care than usual PC but less than specialist care (Quote 16). The third criterion was determined by the ComSA-BPS-RS and/or clinical assessment.

#### 4.2.1. Change Strategy: Partnering with RHS to Create Supporting Infrastructure and Pathways in the Delivery System

Change strategy was required to identify, persuade, and direct patients to be empaneled with ComSA-PCMH, as they were currently receiving care from RHS-based institutions, and unfamiliar with the new care model (Section 2).

As an implementation partner, the central-region RHS played a crucial role in identifying and referring the target population to ComSA-PCMH (Quote 17). To identify eligible patients, a dedicated referral management team was introduced to case find and risk screen patients in the RHS (Quote 18). Then, the RHS clinicians assessed these patients and referred them to ComSA-PCMH if they were suitable and agreeable. In addition, ComSA-PCMH and the RHS provided shared care to empaneled patients still needing specialist care.

To improve patients’ receptiveness to the innovation, RHS-based subsidies (e.g., subsidized medications) were extended to ComSA-PCMH patients. Additionally, special arrangements were made to allow referred patients to easily return to their medical specialist(s) if necessary (Quote 19).

#### 4.2.2. Initial Perception of RHS Implementation Partners: Characterized by “Adoption”

Two key themes described the initial perception of RHS implementation partners: (i) perceived value of the innovation; and (ii) perceived burden in implementing change strategies. Both affected the adoption of the innovation by implementation partners, including the formation of favorable or unfavorable attitudes towards the innovation, experimentation with and/or adaptation of the change strategies.

Implementation partners perceived ComSA-PCMH by comparing it to the usual PC based on cost, convenience, and quality of service delivered to patients. Cost to patients was perceived to be higher than usual PC. Nonetheless, ComSA-PCMH had relative advantages, in terms of better geographical accessibility, availability of an integrated CM service, experience in aged care (Quote 20), option to have shared care with specialists, access to RHS-based subsidies in the community (Quote 21), and an appointment-based system. Perceived value could have been influenced by the way information about the innovation was communicated to the implementation partners. Those who communicated directly with ComSA-PCMH implementers were more likely to appreciate the value of this care model, adopt the innovation, and refer patients to ComSA-PCMH. Otherwise, clinicians tended to regard ComSA-PCMH as “just another collaboration” and lacked the motivation to assess and refer patients (Quote 22).

The day-to-day implementation of ComSA-PCMH was perceived to be burdensome in two areas: (i) systematic case finding of target population using age and postcode, which was thought to be labor-intensive but producing low yields (Quote 23); and (ii) risk stratification using the 37-item ComSA-BPS-RS, which was deemed too long and the eligibility criteria (i.e., highest two risk categories) too restrictive for operationalizing empanelment. However, implementation partners agreed that selectivity was strategically necessary because ComSA-PCMH was resource-intensive thus it would be more cost-effective to reserve care for complex needs patients (Quote 24). To improve adoption among implementation partners, some strategies deemed burdensome were adapted. For example, ComSA-BPS-RS was experimented with briefly and eventually replaced by implementation partners’ pre-existing risk stratification tools (Quote 25).

### 4.3. Innovation III. Shared Care with Local Private GPs for Complex Chronic Patients

The third innovation was shared care with local private GPs for complex chronic patients. The aim was for the PC services to provide complementary services to retain patient care in the community. For example, ComSA-PCMH might offer CM services to GPs’ existing patients with psychosocial care needs while GPs who had their practices opened during evenings and weekends might offer after-hours care to ComSA-PCMH patients (Quote 26).

#### 4.3.1. Change Strategy: Outreach to Local Private GPs

In the first two years of implementation, targeted outreach was made to GPs through phone calls, personal visits by the ComSA-PCMH chief physician, lunchtime networking sessions, and seminars (Quote 27).

#### 4.3.2. Initial Perception of Private GPs: Characterized by Adoption of ComSA-PCMH

Findings are based on initial impressions of ComSA-PCMH by two private GPs in Whampoa. One GP was aware of ComSA-PCMH’s intent to target patients with more complex needs, through direct interactions with ComSA-PCMH’s chief physician and attending networking sessions. However, there was a misconception about ComSA-PCMH being a free clinic for low-income patients (Quote 28). The other GP had no direct interactions with ComSA-PCMH and was unaware of it. This GP was of the opinion that ComSA-PCMH could be complementary to GPs if it was providing social or rehabilitative care, while the provision of medical services by ComSA-PCMH were perceived to pose competition to private GPs and replicate public polyclinic services (Quote 29). Both GPs did not understand the functions of the CM service.

### 4.4. Lessons Learnt

Two emerging themes were identified for lessons learned during the initial implementation of the innovation: i.e., working with complexity and working with the novelty of the innovations (Figure 2).

To work with complex innovations with a high number of soft peripheries, three key ingredients are needed: (i) time—needed for experimentation, negotiations, and adaptations of various adaptable components by implementers; (ii) leadership—needed for facilitating and providing a clear direction for the assimilation process, which could otherwise be inconclusive; and (iii) a simple, reliable, and standardized method for guiding complex decisions for risk stratification and empanelment.

Innovations in their early implementation were still novel to the users due to low observability and trialability. To work with novelty, three further key ingredients are needed: (iv) time—needed by implementers to do outreach activities in addition to direct patient care; (v) direct communication with the implementers—needed to improve partners’ understanding of ComSA-PCMH; and (vi) awareness-building initiatives—needed for understanding less-known service designs, such as CM and empanelment.

## 5. Discussion

### 5.1. Novelty of PCMH as a Care Model and Its Diffusion

This study unpacks the implementation of ComSA-PCMH, a novel care model in Singapore, using theory on diffusion of innovations. As one of the first PCMHs in Singapore, the creation of ComSA-PCMH was primarily a community ground-up effort. This brought about a different implementation experience than PCMH demonstrations which have been introduced into the delivery system by government agencies. For example, PCMHs are systematically disseminated in the US by dedicated national quality assurance bodies through accreditation and technical assistance in the form of quality improvement coaching [51,52,53]. In Australia, the PCMH (or “health care home”) program is helmed by the Department of Health that rolled it out in ten Primary Health Network regions since 2016 [54]. Compared to these government-led disseminations which are centrally planned, innovations introduced from the ground are often diffused more informally first within peer networks. At times, central agencies can play a vital role as an enabler or facilitator [25]. In the case of ComSA-PCMH, the implementers were pathfinding and resolving contextual challenges in partnership with a central agency (i.e., an RHS). This enabled some creative and pragmatic strategies to develop a care model that was both distinct from usual PC models and integrated with the delivery system. First, it empaneled only patients whose care should remain in the community but be unmet by the usual PC. This strategy differentiated its target patient segment from patient populations in usual PC and specialist care. It was also compatible with the Ministry of Health’s national strategies to shift care from hospital to community and to close service gaps in the usual PC in providing quality, community-based care for complex needs patients [55]. Second, ComSA-PCMH reduced the payment gradient between the subsidized RHS clinics and the new care model by adapting the pre-existing RHS-based subsidies. If patients had to pay substantially more when transferring their care to ComSA-PCMH, this care model might not attract patients. Although this payment reform was small-scale and adaptive, it presented an opportunity to explore better financing mechanisms for high-need patients to receive care in the community. Evidence suggests that primary care financing mechanisms that modify the traditional fee-for-service model or replace it with pay-for-performance, bundled, or capitated payments, can incentivize multidisciplinary team-based, person-centered care in the PCMHs [56,57].

While empanelment is a good PC practice that enables the provision of first-contact access, as well as continuous, coordinated, and comprehensive care [41], it is still a novelty in the PC sector in Singapore, and thus, is not commonly understood. Our findings show that it was challenging for other service providers in the delivery system to adopt this innovation. Without adequate outreach efforts, private GPs serving the Whampoa community at large may still regard ComSA-PCMH as a competitor. Meanwhile, even implementation partners tended to directly compare ComSA-PCMH to usual PC models. Such initial perception might have inhibited the characterization of ComSA-PCMH as a valuable new care model that is different from the usual PC. In fact, implementation challenges due to novelty were also faced by other new community-based services in Singapore [58]. As the innovation matures and adoption improves, empanelment may become routinized in the delivery system without requiring dedicated efforts for case finding, risk stratification, and referral management.

Despite a lack of understanding of the care model, there was consensus among implementers and implementation partners that ComSA-PCMH, as a higher-resourced program, should be reserved for more complex patients. This is congruent with the evidence about PCMHs yielding more cost savings and clinical improvements in patients with the most complex needs [59]. However, there have been different definitions of the nature and threshold for complex needs. Indeed, “complex needs” is an emerging term in health services that have inconsistent interpretations [60]. In Singapore, there has been a lack of a standardized method for defining complex needs in chronically ill patients [61]. Efforts have been underway to define patient needs by segmenting the population based on routinely collected medical and healthcare utilization indicators [62,63]. As routinely collected data lack functional and social health indicators crucial for measuring complex needs, additional tools have also been developed to incorporate them, but they often have to be administered by clinicians [64]. ComSA-BPS-RS was developed and locally validated to define complex needs based on all bio-psycho-social dimensions. It can be administered by trained surveyors instead of clinicians [65]. However, our findings show that its adoption was low due to the perceived burden. For better efficiency and equity in resource allocation, standardized stratification or segmentation tools that are simple and reliable are needed to correctly identify intended populations for innovation.

### 5.2. Complexity of PCMH Transformation and Its Assimilation

PCMH transformation is known to be complex and time-consuming, involving radical changes in structures and systems which have to be acquired by multiple players [66]. This corresponds to the theory that assimilation is a complex process involving multiple decisions jointly made by multiple players [25]. The initial teething issues encountered by the implementers in transforming care toward a team-based structure was reflective of assimilation. Such a process resonated with the experience of early adopters of PCMHs in other countries, which suggests that transformation needs to start from a shift in practice and policy perspectives, accompanied by sufficient resources, good leadership, and time [67,68,69]. The literature also emphasizes the importance of applying cultural change strategies for getting whole-staff engagement, shifting practice perspectives, and inculcation of self-reflection on PCMH care philosophies [70,71]. The implementers’ experience resonates with how assimilation is commonly experienced, i.e., “‘complex”, “messy”, and “non-linear” [25]. Challenges faced in assimilation were explained by the theme of “soft peripheries”, which according to the theory is inherent to innovations. It points to the importance of allocating focal and conscious attention from organizational leadership to support efforts to experiment, negotiate, and adapt when innovation is at its experimental phase [50].

ComSA-PCMH was a complex innovation that was continuously evolving, situated within a delivery system that was also continuously evolving. Multiple perspectives were common in the initial experience and perception, for example, the different emergent perspectives by implementers on the operational definitions of complex needs and the self-organizing behaviors by different implementation partners in adopting or abandoning risk stratification tools. These findings point to the realities of working in complex systems, which comprise many “diverse, interdependent and semi-autonomous actors” whose self-organization and interaction with each other may produce non-linear trajectories in the system [72]. This echoes the calls of implementation scientists to shift perspective from seeing implementation as stepwise, linear processes to one that pays attention to both the risks and opportunities in multiple perspectives, emergent causalities, and nuanced pluralities [73,74,75]. Therefore, the sustainability of innovation is less about achieving routinization but more about the ability of the innovation to continuously develop, or evolve [76].

## 6. Conclusions

Using a grounded theory approach, this study unpacked the complexities in change strategies, initial experience and perception, and lessons learned from implementing a new PCMH in Singapore. It is the first study that links early PCMH implementation with theory on diffusion of innovations. Without a theoretical lens, certain implementation processes may be perceived as mere challenges or barriers (e.g., assimilation may be experienced as a disagreement between implementers). With the theory, they can be understood as pertinent parts of the implementation. The study also fills in the literature gap about the implementation of a new PCMH in an Asian context. As an early adopter of the care model, ComSA-PCMH experimented, negotiated, and adapted change strategies based on pragmatic considerations to overcome both country-specific contextual challenges and the inherent complexities of innovations in the care model. Assimilation and adoption were identified as the challenging steps during early implementation, as they involve complex processes participated by multiple players who might exhibit less predictable, self-organizing behaviors. This study contributes to practice and policy considerations by identifying crucial ingredients for PC transformation, including time, leadership, standardized methods, direct communication, and awareness building for the innovations.

This study was limited by the application of the single-method, though that was complemented by analysts’ ethnography-like experience. The evolution of the innovations was not fully captured due to a cross-sectional study design, limited contact with the key informants, and a lack of participation from other stakeholders in the delivery system such as other PC and community service providers. We recommend further research to examine four areas: (i) the later stages of implementation; (ii) the development of care management models; (iii) strategic partnerships for PC innovations; and (iv) organizational readiness for complex implementation, using multiple methods in a longitudinal fashion.

## Figures and Tables

**Table 1:** Exemplary quotes from the study.

Category—Theme/Code		Exemplary Quotes
Innovation I. Team-Based Care Delivering Integrated Medical and Psychosocial Services		
1	Change strategies—repurposing TF’s pre-existing services and infrastructure	“…the idea of having a comprehensive primary care that serves the needs of the patients and his caregivers and to integrate the care between outside providers as well as internal colleagues, is similar. The idea of developing a PCMH is similar (to existing TF services).” (Implementer #11)
2	Change strategies—integration between PC clinic and CM service	“...to integrate them and make sure that the patients that is cared for by CM and the clinic can actually have a seamless coordination of care without having some care components falling through… working out how each of the different components work together… so there are the different work processes that we are learning as we operationalize the whole PCMH… we have morning huddles—where we actually have a quick word with each other twice a week… and we attend interdisciplinary meetings.” (Implementer #11)
3	Change strategies—service redesign based on local needs	“In the beginning, when we are still looking at rental houses (i.e., lower-income neighborhoods), you still know they have family but they are left alone, on their own. But right now, the clientele that we are seeing in the past one and a half year, I think we are seeing a lot more with families.” (Implementer #22)
4	Negotiations—ComSA-PCMH should be reserved for patients with highest needs (example 1)	“This clinic is resource intensive, (so) we should not see low risk patients. Otherwise, no value because the GPs and polyclinics are doing a great job in that. However, I think (other implementers) want to see (patients) earlier. I think they want this clinic to be like a real clinic, across life-course.” (Implementer #21)
5	Adaptations—eligibility criteria revised (example 1)	“Initially we put at 4 (ComSA-BPS-RS risk category), then drop to 3, then drop to 2.” (Implementer #21)
6	Negotiation—different perspectives on the need for ComSA-PCMH to provide home-based care (example 2)	“I feel that, when you deal with frail elders, the primary care has to be more flexible than this. I know where they (i.e., implementers with different perspective) come from. They are coming from a place of financial sustainability (while) I come from a person-centered perspective.” (Implementer #21)
7	Experimentation—ComSA-PCMH physician providing home medical service without supporting funding (example 2)	“The home visit is not covered by the government, they don’t subsidize, I can’t charge him (the patient) for the home visit, because he has no means to pay and it will disengage, it will make things worse. So the only thing I can do is to go do a home visit.” (Implementer #02)
8	Adaptations—a new home medical program was introduced (example 2)	“Now for home care in Whampoa, a new home medical service (would) go and see (the patients)... The doctors don’t do home visit, the (clinic) nurse don’t do home visit. Then the care managers—there’s a bit of tweaking. Now care managers can do direct nursing care. By right also cannot, care manager strictly no touching.” (Implementer #21)
9	Initial experience—assimilation characterized by multiple perspectives	“Everybody had their own way of working, their own ideas, they came with different baggage right, because they are all from different walks of professions. So to put them together and, I think also, ComSA-PCMH is a work in progress, it’s evolving.” (Implementer #11)
10	Initial experience—assimilation characterized by soft peripheries	“They (implementers) need to talk to each other, be comfortable negotiating about their role. Because they need some clarity of what each is supposed to do, but there’s a lot of blurring in the middle, you kind of just have to figure out what’s the best way of doing what.” (Implementer #13)
11	Barrier to assimilation—perceived lack of leadership in decision-making	“Nobody really know you must go this way or not… so it was confusion because there wasn’t clear leadership…” (Implementer #11)
12	Barrier to assimilation—slow progress in transforming the clinical information systems	“The information technology basically still functions in silo... So everyone uses their own little components, you know. But there is no function... there’s no structure, so communication is happening in an unofficial way, which is very difficult to keep track.” (Implementer #11)
13	Facilitator for assimilation—intrinsic motivation to contribute to aged care	“My impression of them (i.e., the implementers from TF) is that they are very passionate about their work. So lots of energy and enthusiasm to do more and do better.” (Implementation partner #18)
14	Facilitator for assimilation—relevant competencies and/or training	“the (ComSA-PCMH) model’s different, and the competencies required is actually higher. And we almost cannot hire people with that kind of training background, experience nor certainly not people with that kind of mindset required in the service model… So when you hire somebody, you have to really train them up to the competencies of the model, while getting them to understand why we chose to do things the way we do” (Implementer #13)
15	Facilitator for assimilation—observability of impacts from invested efforts	“I think the direction is clearer (after 1.5 years), and people could see their contributions. Then, they would, they work happier because they know that what they’re doing is of value. And that it’s not so frustrating you know.” (Implementer #11)
Innovation II. Empanelment of a Specific Population		
16	Innovation—targeting patients in a specific risk stratum between primary and specialist care (whose needs are unmet by usual PC)	“…you need some provider who can interface with the hospital and the community. Because the rest of the community providers, particularly the doctors like the GPs or the polyclinics, are not yet at the level where they want to provide the kind of care that older people—especially the frail, complex ones—that they really need. So until such time, you need a more specialized unit that sits between the hospital and community that can really provide intervention at a population level as well as at the individual-care level by looking at catalyzing health systems and connecting all the dots between hospitals, providers and the people.” (Implementer #13)
17	Change strategies—partnering with RHS to create supporting infrastructure and pathways in the delivery system	“ComSA was then a service provider partner, in this whole setup. So, I guess then one can see that the collaboration grew from not just the support of subsidized medications but that of actually the RHS hosting the infrastructure in terms of holding the tenancy for the building where they were sited, and along with the various factors such as enabling factors such as finance, flow of patients.” (Implementation partner #12)
18	Change strategies—processes involved in referral management	“One of the executives will churn out a list of patients that fulfil the (eligibility) criteria. This list is then sent to one of our care facilitators, who then… prompts the doctor that when the patient arrives, discuss ComSA-PCMH referral with the patient… And then the patient gets transferred to one of our care facilitators... What she then does is to dive into greater detail about the COMSA clinic… And then she will set up appointments if patient’s agreeable… then we will create a referral letter and send it across.” (Implementation partner #18)
19	Change strategies—options for patients to return to specialists if necessary	“If there’s anything (that warrants patient care to be transferred back to the hospital), let’s say patient condition worsens or what, there’s this telecommunication that the doctors there (in ComSA-PCMH) and our doctor (in the hospital) can communicate to update each other whether there’s a need to come back or what or, you know.” (Implementation partner #17)
20	Perceived value—comparison to usual PC (ComSA-PCMH more experienced in aged care)	“But again the level of care between GP and ComSA-PCMH (can) provide might be very different. ComSA-PCMH does provide a more holistic kind of care to patients. That’s why we see value and we see that in fact, they may feel safer if our geriatric patients are cared for by ComSA-PCMH.” (Implementation partner #16)
21	Perceived value—comparison to usual PC (access to RHS-based subsidies)	“So the advantage of this is that... ComSA-PCMH would then be receiving medications at that price which typically no non-RHS primary care provider would normally receive it at.” (Implementation partner #12)
22	Perceived value—influenced by opportunity for direct communication with implementers	“So, staff who knew about this service early on, knew that it was a service meant for patients who had psychosocial challenges. They knew that they would be able to fill a service gap that the polyclinic has problems trying to fill, so they were much more happy to, and receptive towards referring patients who would fulfil their criteria. However, in current state where we inform the doctors that this is another collaboration, then to them it is another collaboration. Just that the referral criteria, the inclusion criteria is somewhat different. So they may not be able to understand the true meaning and the intentions and the vision and mission that the ComSA clinic has… So they may just look upon it as a business as usual, rather than something that may value add to the patient.” (Implementation partner #18)
23	Perceived burden—systematic case finding was labor intensive and producing low yield	“In one month, I pull (i.e., case find) 40 plus (potentially eligible patients). But out of the 40 plus—this is just a rough number—maybe I already taken out, like, 10 because of various reasons: maybe they are still not stable or they haven’t concluded their diagnosis... Yeah then after that there’s patient choice (i.e., that patients might not agree to be referred) and all that… So you really have to funnel, funnel, funnel. Then the number will not be a lot.” (Implementation partner #14)
24	Perceived value—ComSA-PCMH was resource intensive hence should be reserved for complex needs patients	“If we have abundant resources, then we can shift the (ComSA-BPS-RS risk category) cut-off for delivering that service at a lower ComSA-BPS-RS score (i.e., lower-risk patients). But if I were to tell you that we are very scarce in terms of resources, then we need to shift the ComSA-BPS-RS cut-off very high. …hence, the point about sustainability lah. I don’t think that’s a question about whether you should or should not have this service. The question then really is who do you deliver this service for, given the resources you have.” (Implementation partner #18)
25	Perceived burden—replacing ComSA-BPS-RS with biomedical markers	“So patients who would be seen and followed up by ComSA-PCMH were those who were ComSA-RS positive plus medical problems. But we basically told them (i.e., TF implementers) that, for us to try and do the ComSA-BPS-RS for you… (it) would be challenging because there are very few patients who will satisfy the ComSA-BPS-RS positive criteria. So we actually said, we just use biomedical markers, is that okay? So they eventually said okay.” (Implementation partner #18)
Innovation III. Shared Care with Local Private GPs for Complex Chronic Patients		
26	Innovation—shared care with private GPs for complex chronic patients	“Older patients are very complex, and some of the GPs may not have been practicing that way, and there may be some knowledge gap, I would think, in terms of taking care of older people… a lot of things are just social problems and they (i.e., the private GPs) can’t take care of their social problems… (while) ComSA-PCMH has coverage (for complex needs patients), but we’re not open (on weekends and evenings). So, one GP was very happy to see them during those hours, but he feels like it may be better to work with us so we can share the care. We take on the ongoing, long term kind of like, care for the chronic diseases and the social problems and all that. But they’re happy to see them for the ad-hoc, acute things, and then there’ll just be communication.” (Implementer #13)
27	Change strategies—targeted outreach to private GPs	“Certainly the previous chief physician had more than done their fair share of making their rounds (in) the (private) GPs in the Whampoa neighborhood. They have organized what they term as brown bag engagement sessions, where they just buy simple lunch, unlike the buffet spread that people, drug companies offer. But what they make up for in lack in food they make up for with sincerity.” (Implementation partner #12)
28	Initial perception—understood ComSA-PCMH’s intent to provide complex care needs but misunderstood its care provision as free	“I think they have done a good job you know, taking care of the elderly in this area… You know, offering free services to them.. rehabilitation and management chronic diseases and I think that’s a good outlet for the people here to go to. I think they are quite comprehensive in the care of the elderly.” (Private GP #1)
29	Initial perception—perceived complementarity of ComSA-PCMH services to private GPs’ in social care only	“We are getting more and more dementia patients. And that’s very hard for us (i.e., private GPs) to manage. So that’s where you all (i.e., ComSA-PCMH) have the time and the facilities to get them (i.e., the patients), get together, mix together, and socialize together. That’s important. Because a lot of them are lonely you see. So that’s what community center is about, and you facilitate that. And then you give… erm… You have the gym, you have the dancing classes, that’s useful, and maybe once in a while have a visiting physiotherapist, something like that. That’ll be useful. But to have clinics to take over from GPs… I don’t think GPs will be happy with that… we don’t refer at the same level (i.e., to physicians in ComSA-PCMH). We refer to specialists. You know what I mean?” (Private GP #2)

## Data Availability

The data presented in this study are not publicly available due to the privacy rights of the participants, in accordance with the informed consent of this study.

## Supplementary Materials

The following are available online at https://www.mdpi.com/article/10.3390/ijerph182111160/s1, Supplementary File S1. Semi-Structured In-Depth Interview Topic Guides.
