# Supplementary material for: Early Implementation of a Patient-Centered Medical Home in Singapore: A Qualitative Study Using Theory on Diffusion of Innovations
Source: Int J Environ Res Public Health. 2021 Oct 24;18(21):11160. doi: 10.3390/ijerph182111160 (PMC8583400; doi:10.3390/ijerph182111160)
Supplement: Supplementary file 1 [file ijerph-18-11160-s001.zip › ijerph-1429508-supplementary.pdf]

Supplementary File 1. Semi-Structured In-Depth Interview Topic Guides

<u>Interview Topic Guide 1 – Service Providers</u>
Eligibility criteria: at least 3 months of experience interacting with ComSA-PCMH
Objective: To understand the challenges, barriers and facilitators in implementing the delivery-system change from key stakeholders' perspective

(Introduction, describe the purpose of the discussion, and how long it is expected to take; and obtain informed written consent)

Before we start, I would like to remind you that there are no right or wrong answers in this discussion. We are interested in knowing what you think, so please feel free to be frank and to share your point of view, regardless of whether you agree or disagree with what you hear. It is very important that we hear all your opinions.

Your response will be confidential and will not be shared to others outside the study team. Your response will also have nothing to do with your work-related assessment.

(Members of the research team should also introduce themselves and describe each of their roles).

1. Opening questions

1.1. Could you tell us a little about yourself?
<u>Prompt:</u>
- What is your professional background?

1.2. How is your usual day like?
1.3. How long have you been working as a healthcare provider?
1.4. How long have you been working in ComSA-PCMH?
<u>Prompt</u>:
- How did you become a healthcare provider in this clinic?
- Who recruited you?
- How was the process?

1.5. Please tell me about your job.
<u>Prompt:</u>
- Roles and responsibilities
- Average days per month work; average hours per day work

1.6. How do you think about the workload that you have as the healthcare provider (refer to each profession, according to the participants)?
<u>Prompt:</u>
- How could it be improved/maintained?
- What are some challenges and barriers you face in your work?
- How would you usually address those challenges and barriers?
- How do you think your supervisor or colleagues can help you address those challenges?

1.7. How was the training on the job done?
<u>Prompt:</u>
- What kinds of trainings were you get, related to your job scope?
- What are the topics?
- Who conducted the training?
- How frequent are the training done?

1.8. How would you describe the training that was provided?
<u>Prompt:</u>
- What did you learn, and what did you not learn?

- How useful have these been to your work?

1.9. How was the supervision done for your work?
<u>Prompt:</u>
- Who do you report to?
- Who routinely supervises and supports your work?
- What do you think of the supervision?
- What benefits do you get?
- What new or additional things have you begun to do as a result of the training, support, and supervision?

2. Practice transformation

2.1. Please tell me what you remember about the care delivery before the ComSA-PCMH was built.
<u>Prompt</u>:
- Before November 2016

2.2. Please tell me how the care delivery has changed after the ComSA-PCMH was built
<u>Prompt:</u>
- What was happening in the bigger picture?
- What triggered the change?
- Describe the internal and external motivators relative to the patient-centered care model (may be positive or negative)
- Describe the change process, particularly in relation to the practice change and development model.
- What were the challenges and barriers for the change process?
- What were the facilitators for the change process?
- How was the buy-in from practice leadership and staff?
- How has leadership facilitated/hindered the process?
- Describe any relationship patterns among key stakeholders.

2.3. What is the evidence for a transformation among the individual staff?
<u>Prompt:</u>
- How did the transformation affect your roles?
- How did the transformation affect your vision?
- How did this unfold?

2.4. What is the evidence of transformation at the practice level?
<u>Prompt:</u>
- Did it move toward becoming a learning organization?
- How did this unfold?

2.5. How is your general perception of the practice transformation?
<u>Prompt:</u>
- How did it change your interaction and work with your co-workers?
- How did it change your interaction and work with you patients?
- How did it affect the patient care and other outcomes?

2.6. What do you think are the strengths and areas for improvement?
<u>Prompt:</u>
- Which are the areas where there has been significant change and what are factors responsible for this?
- Which are the areas where there has been the least change and what are factors responsible for this?
- How helpful are these changes have been?

3. Patient-provider partnership and communication

3.1. How would you describe your communication with the patients?
Prompt:
- What kind of information you gave about the patient's condition when you treat the patient in a day-to-day basis?
- What kind of information you gave about the patient's medication?
- What kind of information you gave on the self-management or follow-up care at home for the patient?
- What kind of information you gave on the social support for the patient?
- What other information you gave on the resources that the patient could use to manage their condition?

3.2. Please explain how and where you meet the patients for their care delivery.
Prompt:
- At the clinic
- Home visits
- Community meetings
- Small group meetings
- Other ways?

3.3. Please explain how you communicate with patients for their care delivery.

4. **Comprehensiveness**

4.1. What is the approximate number of patients that you look after?
Prompt:
- How was the patient recruited?

4.2. What kind of services are available for patients (40 years and older) in this clinic/care management?
Prompt:
- Physical care
- Mental care
- Social care

4.3. How was the uptake of the services?
Prompt:
- What factors enable the patients to receive these services?
- What factors may prevent the patients from receiving these services?
- What are some reasons why some patients not getting/do not want the services?

4.4. What other services that you provide in the clinic/care management?
Prompt:
- What services that you provide in partnership with other institution/practice outside ComSA-PCMH?

5. **Coordinated care/care management**

5.1. How would you describe the care coordination process?
Prompt:
- How do you usually make referrals for your patient?
- How do you usually arrange for appointment for visits in different places the patients go (e.g. specialist clinic, acute hospitals, social care, etc.)
- How do you usually exchange medical information/histories of your patients with other health providers?
- How do you usually follow-up medical information/histories of your patients with other health providers?
- What kind of documentation do you usually keep for the medical record?
- How the transition of care was coordinated between the care providers?

- What were the challenges and barriers during the process?
- What do you think the effects of the coordinated and team based care?

5.2. What do you think of supervision on the care management and coordination?

6. Enhanced access

6.1. What is the usual work hour for the clinic and/or care management program?

Prompt:

- What kind of care do you provide during weekend, public holiday, and after office hours?
- What kind of information were given to the patients about places to go during weekend, public holiday, and after office hours?
- How do you usually provide care for the patients if they need emergency care?

6.2. What modes of communication do you usually use with your patients?

6.3. How was your experience in appointment booking allocation in the clinic?

Prompt:

- What are the challenges, barriers, and facilitators?
- How long is usually the waiting time to get consultation?
- How likely is it to have the same day appointment for the patients? Why?

7. Partnership with other primary care providers

7.1. How would you describe the partnership of the clinic with other primary care providers?

Prompt:

- How do you think their perception of the care model/delivery done by ComSA-PCMH?
- How would you describe their willingness to refer their patients to the clinic?
- What do you think the reasons why they are/are not willing to refer their patients to the clinic?
- What are the challenges and barriers during the process?

7.2. What is your general perception on this partnership with other primary care providers?

Prompt:

- What do you think the positive and negative impact of it?
- How could we improve the partnership between the primary care providers?

8. Impacts

8.1. What changes do you see among the participants in the health-related behaviors, utilizing health services?

Prompt:

- Which are the areas where there has been significant change, and what are the factors responsible for this?
- Which are the areas where there has been the least change, and what are the factors responsible?

8.2. How would you describe the changes in the participants' health after they receive care from the clinic and/or care management?

Prompt:

- What do you think contributed to those changes?

8.3. What went well in the clinic care delivery, in your opinion? Why?

8.4. What recommendations would you have to improve this clinic/care management care delivery?

Prompt:

- What would enable you to be able to work effectively?
- What aspects of the clinic/care management would you like to see improve?

9. Closing question

9.1. Before we wrap up, would you have any final thoughts that you would like to share?

This brings us to the end of our interview for today. Thank you once again for sharing with us today. We appreciate you time, and the study will benefit greatly from al the inputs that you have shared. Should you have any further questions, feel free to contact us.

<u>Interview Topic Guide 2: Management staff</u>
Selection criteria: at least 3 months of experience working with ComSA-PCMH
Objective: To understand the challenges, barriers and facilitators in implementing the delivery-system change from key stakeholders' perspective

(Introduction, describe the purpose of the discussion, and how long it is expected to take; and obtain informed written consent)

Before we start, I would like to remind you that there are no right or wrong answers in this discussion. We are interested in knowing what each of you think, so please feel free to be frank and to share your point of view, regardless of whether you agree or disagree with what you hear. It is very important that we hear all your opinions.

You probably prefer that your comments not be repeated to people outside of this group. Please treat others in the group as you want to be treated by not telling anyone about what you hear in this discussion today. Let's start by going around the circle and having each person introduce herself.

(Members of the research team should also introduce themselves and describe each of their roles).

2. Opening questions

9.2. Could you tell us a little about yourself?
Prompt:
- What is your professional background?

9.3. How is your usual day like?
9.4. How long have you been working as a healthcare provider?
9.5. How long have you been working in ComSA-PCMH?
Prompt:
- How did you become a care manager?

9.6. Please tell me about your job
Prompt:
- Roles and responsibilities

9.7. How was the training on the job done?
Prompt:
- What kinds of trainings were you get, related to your job scope?
- What are the topics?
- Who conducted the training?

9.8. How would you describe the training that was provided?
Prompt:
- What did you learn, and what did you not learn?
- How useful have these been to your work?

9.9. How was the supervision done for your work?
Prompt:
- Who do you report to?
- Who routinely supervises and supports your work?
- What do you think of the supervision?

- What benefits do you get?
- What new or additional things have you begun to do as a result of the training, support, and supervision?

10. Practice transformation

10.1. Please tell me what you remember about the care delivery before the physical clinic in Whampoa was built.

Prompt:

- Before November 2016

10.2. Please tell me how the care delivery has changed after the physical clinic in Whampoa was built

Prompt:

- How is this different from 6 months ago?
- How has the practice changed in the last 6 months?
- What was happening in the bigger picture? How did it happen?
- Which components of the new model of care are in place in the practice?
- How did they get in place?
- Which components are not in place and why?
- How did the transformation affect the scheduling and access arrangements?
- How did the transformation affect the care delivery process?
- How did the transformation affect the practice management?
- How did the transformation affect the information system and technology?

10.3. Describe the internal and external motivators relative to the practice change towards patient-centered care model (may be positive or negative)

Prompt:

- Describe any changes in your external environment over the last 6 months (e.g. rules and regulations, insurance/payment system, healthcare system, etc.)
- How these affect the practice transformation?
- Describe any changes in your internal environment over the last 6 months that affect the practice transformation.

10.4. How would you describe the practice ability to (effectively) deal with the ongoing demand of change?

Prompt:

- Describe the change process, particularly in relation to the practice change and development model/pathways.
- How was the practice change translated to a day-to-day operational flow?
- How were the staff understanding the change process?
- How was the buy-in from practice leadership and staff?
- How has leadership facilitated/hindered the process?
- How was the relationships and communication among key stakeholders during the practice change process? Describe any relationship patterns among key stakeholders.
- What were the strengths and areas for improvements?
- What were the challenges and barriers?

10.5. What is the evidence for a transformation among the physicians?

Prompt:

- How did the transformation affect the physician and staff roles?
- How did the transformation affect the physician and staff vision on them?
- How did this unfold?

10.6. What is the evidence of transformation at the practice level?

Prompt:

- What is the evidence of the paradigm shift of the practice towards the patient-centered care?

- Did it move toward becoming a learning organization (with sharing and ongoing learning for continually improving patient-centered care)?
- How did this unfold?

10.7. How is your general perception of the practice transformation?

Prompt:

- How did it change your interaction and work with your co-workers?
- How did it change your interaction and work with you patients?
- How did it affect the patient care and other outcomes?

10.8. What do you think are the strengths and areas for improvement?

Prompt:

- Which are the areas where there has been significant change and what are factors responsible for this?
- Which are the areas where there has been the least change and what are factors responsible for this?
- How helpful are these changes have been?

11. **Comprehensiveness**

11.1. What kind of services are available for patients (40 years and older) in this clinic/care management?

Prompt:

- Physical care
- Mental care
- Social care

11.2. How was the uptake of the services?

Prompt:

- What factors enable the patients to receive these services?
- What factors may prevent the patients from receiving these services?
- What are some reasons why some patients not getting/do not want the services?

11.3. What other services that you provide in the clinic/care management?

12. What services that you provide in partnership with other institution/practice outside ComSA-PCMH?

13. **Coordinated care/care management**

13.1. How would you describe the care coordination process?

Prompt:

- How do you usually make referrals for your patient?
- How do you usually arrange for appointment for visits in different places the patients go (e.g. specialist clinic, acute hospitals, social care, etc.)
- How do you usually exchange medical information/histories of your patients with other health providers?
- How do you usually follow-up medical information/histories of your patients with other health providers?
- What kind of documentation do you usually keep for the medical record?
- How the transition of care was coordinated between the care providers?
- What were the challenges and barriers during the process?
- What do you think the effects of the coordinated and team based care?

13.2. How would you describe the supervision on the care management and coordination?

14. **Enhanced access**

14.1. What is the usual work hour for the clinic and/or care management program?

Prompt:

- What kind of care do you provide during weekend, public holiday, and after office hours?

14.2. How would you describe the appointment booking allocation in the clinic?

Prompt:

- What are the challenges?
- What are the barriers
- What are the facilitators?
- How long is usually the waiting time to get consultation?
- How likely is it to have the same day appointment for the patients? Why?

15. Partnership with other primary care providers

15.1. How would you describe the partnership of the clinic with other primary care providers?

Prompt:

- How do you think their perception of the care model/delivery done by ComSA-PCMH?
- How would you describe their willingness to refer their patients to the clinic?
- What do you think the reasons why they are/are not willing to refer their patients to the clinic?
- What are the challenges and barriers during the process?

15.2. What is your general perception on this partnership with other primary care providers?

Prompt:

- What do you think the positive and negative impact of it?
- How could we improve the partnership between the primary care providers?

16. **Context adaptation and attention to the fitness landscape**

16.1. Describe how the practice stays in touch with the community.

16.2. Describe how the practice seeks to influence to community

Prompt:

- What marketing efforts is the practice pursuing (e.g. advertising)?
- Who is the target?

16.3. Describe how the practice stays in touch with the rest of the healthcare system

16.4. Describe how the practice seeks to influence the health care system

16.5. What went well in the clinic care delivery, in your opinion? Why?

16.6. What recommendations would you have to improve this clinic/care management care delivery?

Prompt:

- What would enable you to be able to work effectively?
- What aspects of the clinic/care management would you like to see improve?

17. Closing question

17.1. Before we wrap up, would you have any final thoughts that you would like to share?

This brings us to the end of our interview for today. Thank you once again for sharing with us today. We appreciate you time, and the study will benefit greatly from al the inputs that you have shared. Should you have any further questions, feel free to contact us.
